# Acute effects of vinegar intake on some biochemical risk factors of atherosclerosis in hypercholesterolemic rabbits

**DOI:** 10.1186/1476-511X-9-10

**Published:** 2010-01-28

**Authors:** Mahbubeh Setorki, Sedighe Asgary, Akram Eidi, Ali Haeri rohani, Majid KHazaei

**Affiliations:** 1 Department of Biology, Science and Research Branch, Islamic Azad University, Tehran, Iran; 2 Isfahan Cardiovascular Research Center, Applied Physiology Research Center, Isfahan University of Medical Sciences, Isfahan, Iran; 3 Department of Physiology, Faculty of Medicine, Isfahan University of Medical sciences, Isfahan

## Abstract

**Background:**

Exaggerated postprandial spikes in blood glucose and lipids induce proportional increases in oxidative stress, which acutely trigger impairment endothelial, inflammation and increased risk of future cardiovascular events. In this research, we have investigated acute effects of vinegar intake on some of the biochemical atherosclerosis risk factors in high cholesterol fed rabbits to see if we can find a probable protective value for it.

**Methods:**

The rabbits were randomly divided into four groups: normal diet, high cholesterol diet (%1cholesterol), %1 cholesterol with 5 ml vinegar (low dose), %1 cholesterol with 10 ml vinegar (high dose). After fasting for 12-15 hours, blood samples were taken to determine baseline values. Three hours after feeding, blood samples were collected again to investigate acute effects of vinegar intake on the measured factors.

**Results:**

Using high-dose vinegar with cholesterolemic diet caused significant reduce in LDL-cholesterol (LDL-C), oxidized-LDL (ox-LDL), malondialdehyde (MDA), total cholesterol (TC) and apolipoprotein B (ApoB) in comparison with hypercholesterolemic diet. Consumption low-dose vinegar with cholesterolemic diet induced a significant decrease in fibrinogen and glucose compared to hypercholesterolemic diet. Level of serum nitrite, nitrate, triacylglycerol (TAG), HDL-cholesterol (HDL-C), apolipoprotein A (ApoA), serum glutamic pyruvic transaminase (SGPT), serum glutamic oxaloacetate transaminase (SGOT) and C-reactive protein (CRP) were not significantly difference in low and high doses vinegar with cholesterolemic diet compared to hypercholesterolemic diet. A significant difference was observed for LDL-C, ApoB100 and TC between low and high doses vinegar.

**Conclusion:**

This study suggest that vinegar, might have some acute effects on biochemical risk factors of atherosclerosis and a probable protective value can be considered for its postprandial use.

## Introduction

Atherosclerosis, a chronic inflammatory response affecting arterial blood vessels, is the leading cause of death in the developed world. Endothelium has an important role in vascular tone regulation and its dysfunction is a key factor in progression of atherosclerosis. Several studies have shown that a transient increase in blood concentrations of triglycerides (TG) and fatty acids can affect endothelium-dependent vasodilatation [1,2]. Considering that after each meal, blood concentrations of glucose and lipids are raised and this postprandial increase lasts for a rather long time, these changes might be of importance in the process of atherosclerosis initiation and progression [3]. It has been also suggested that hypertriglyceridemia and hyperglycemia result in production of reactive oxygen species (ROS) which it leads to the activation of nuclear factor-kappaB [4]. This factor is a transcription factor which is contributed in immunity, inflammation and regulation of cell proliferation, growth and apoptosis by controlling the expression of many genes [5]. These are all among the mechanisms that might be contributed in the progression of atherosclerosis [4]. Patients with higher TG level have had raised concentration of soluble adhesion molecules. Also, postprandial lipoproteins can induce membrane expression of adhesion molecules which might be mediated by the oxidative changes of these particles [6].

Atherosclerosis can prevent and treat with different drugs. Because of length of therapy and vast majority side effects of chemical drugs in treatment of atherosclerosis, herbal medication may be suitable substitute for these drugs. Dietary phenolic compounds, in vegetables and fruits and their juices have shown antioxidant activity which can have positive effect on human health [7] so we examined postprandial effects of vinegar intake on biochemical risk factors of atherosclerosis in high cholesterol fed rabbit.

Vinegar is one of the products of grape. A number of studies have demonstrated that grape juice can decrease cholesterol [8] improve endothelial function [9] and enhancing the resistance to oxidative modification of low density lipoprotein (LDL) [9].

Vinegar which is used commonly as a condiment has been proven to have some medical uses as well. Acetic acid is the main component of vinegar. Some other constituents include, anthocyanins (e.g. Cyanidin-3-glucoside) flavonols (e.g. quercetin, kaempferol), flavanols (Catechin, epicatechin) [10], vitamins, mineral salts, amino acids and nonvolatile organic acids (eg. tartaric, citric, malic, lactic) [11]. Vinegar has shown such multiple effects as enhancement of glycogen repletion [12], prevention of hypertension [13], stimulation of Ca^+2 ^absorption [14] and reduction serum total cholesterol and triacylglycerol in animal studies [15]. Many recent studies have documented that vinegar ingestion decreases the glucose response to a carbohydrate load in normal and diabetic subjects [16,17]. All these data suggests a probable protective value for vinegar. Considering that vinegar is a safe product, widely available and affordable, we studied acute effects of vinegar intake on some of the most important risk factors of atherosclerosis in rabbits fed a high cholesterol diet.

## Experimental methods

### Preparation of the plant

First the genus and species were verified by a botanist (*Vitis Sylvestris*, herbarium number 15810) from the Research Center of Isfahan Province Natural Resources. Then the grapes were collected in Aminabad region of Isfahan and vinegar was produced with traditional methods [18]. In order to standardize the vinegar, some factors such as density, pH, vitamin C, acetic acid, anthocyanin and flavonoids were measured.

### Animals and experimental design

Thirty two male New Zealand rabbits with an average body weight of 1910 ± 257 g were procured from Razi Institute of Iran. The animals were acclimatized under room temperature and humidity with regular light/dark cycle for two weeks and had free access to water and a standard powdered purified diet (Dampars Co, Iran) which consisted of 10% protein, 40-50% carbohydrates, 2% vegetable fat and 15-25% fiber. At the end of this period, rabbits were randomly divided into four groups of eight. Animals were fasted for 12-15 hours and venous blood samples were taken to determine baseline values. After this, each group received one of the four experimental diets: normal diet, high cholesterol diet (%1cholesterol), %1 cholesterol with 5 ml vinegar (low dose), and % 1 cholesterol with 10 ml vinegar (high dose). The cholesterol (1 g for each animal) was dissolved in 2 ml olive oil given to high cholesterol diet animals by oral gavage once. The same volume of olive oil was given to control animals. After cholesterol gavage, the vinegar (5 or 10 ml)was also given orally to animals by oral gavage [19,20]. After 3 hours of the first dose of experimental diets (this period falls within the steady-state period of lipid absorption), again venous blood samples were collected in order to study the acute effects of vinegar [21]. The study was reviewed and approved by the ethics committee of Isfahan University of medical sciences.

### Measurement biochemical factors in rabbit

Blood samples were centrifuged at 3500 rpm for 20 minutes to obtain serum and plasma. The plasma was used for fibrinogen and malondialdehyde (MDA) measurement and the serum for other biomarkers.

Serum total cholesterol (TC), triacylglycerol (TAG), low density lipoprotein cholesterol (LDL-C), high density lipoprotein cholesterl (HDL-C), apolipoprotein A (ApoA), apolipoprotein B (ApoB), serum glutamic pyruvic transaminase (SGPT), glucose, oxaloaetate transaminase (SGOT) and serum glucose were determined using standard enzymatic kits (Pars Azmoon Co, Iran) and auto analyzer (Hitachi 902, Japan). MDA was measured by spectrophotometric method [22]. Oxidized LDL (ox-LDL) (Promokine Co, Germany), C- reactive protein (CRP) (Kamiya Biomedical Co, CRP ELISA Rabbit, USA) were measured using enzyme-linked immunosorbent assay kit according to manufacturer's instructions and fibrinogen was measured using coagulation kit (Mahsayaran Co, Iran). The serum level of nitrite and nitrate were measured using a colorimetric assay kit (R&D Systems, USA) that involves the Griess reaction.

### Measurement physiochemical factors in vinegar

pH was determined by pH meter, density by densitometer, vitamin C assayed by spectrophotometeric method at 520 nm and determined photometrically with 2,4 dinitrophenyl hydrazine to form the red bis-hydrazone which is reduced to a coloreless form [23], total flavonoid content was measured by aluminum chloride colorimetric assay. The absorbance was measured against prepared reagent blank at 510 nm [24], total anthocyanin was assayed by spectrophotometeric method at535 nm [25] and the method used to measure the total acidity of the vinegar is an analytical chemistry technique called an acid-base titration [26].

### Statistical analysis

Results are given as Mean ± SD. Data were analyzed statistically using One-Way-ANOVA test followed by LSD post test. Differences between the baseline values and the values postprandial calculated and then used One-Way-ANOVA for comparing between groups. Then pairwise multiple comparisons were performed using LSD post test. In all instances, p value less than 0.05 was considered significant.

## Results

### Determination of some physiochemical factors in vinegar

After analyzing vinegar factors the amount of vitamin C was 8.02 ± 0.02 (mg/dl), acetic acid percent 15.81% ± 0.04, total anthocyanin in 100 g of vinegar 3.25 ± 1.02 (mg/100 g) and flavonoids in 100 ml of vinegar 1.071 ± 0.06 (g/100 ml equivalent naringenin). The density and pH were 1.042 ± 0.002 (g/cm^3^) and 3.58 ± 0.01 respectively.

### Glucose, lipoprotein and apolipoprotein

In high-cholesterol group, serum glucose levels was increased significantly compared to the normal-diet group (*P *< .0001). Using low-dose vinegar with cholesterolemic diet induced a significant decrease in serum glucose compared to hypercholesterolemic diet (*P *= .007), but the difference was not significant after high-dose vinegar. No significant difference was found between two control groups with regard to TC, TAG, LDL-C, HDL-C, and ApoA1 and ApoB100. Following concurrent use of high-dose vinegar with cholesterolemic diet, ApoB100, TC and LDL-C levels were significantly decreased in comparison with hypercholesterolemic diet group(*P *= .049 *P *= .007, *P *= .027 respectively). Consumption of low-dose vinegar with cholesterolemic diet did not change serum lipoproteins and apolipoproteins levels significantly in comparison with hypercholesterolemic diet. Similar results were observed for TG, ApoA1 and HDL-C when compared between high cholesterol diet group and high-dose vinegar with cholesterolemic diet group. Significant differences were observed between low and high-dose vinegar groups by TC, LDL-C, ApoB100 (*P *= .013, *P *= .025, *P *= .038 respectively). ApoA1 concentration was increased in low-dose vinegar with cholesterolemic diet group compared to rabbits fed high cholesterolemic diet, but the difference was not significant (*P *= .057) (Table 1).

**Table 1:** Comparison of glucose, lipoproteins and apolipoproteins values before (baseline) and after (postprandial) experimental diet

Biochemical factors(mg/dl)		Groups			
		**Cholesterolemic diet**	**5 ml Vinegar with %1 chol**	**10 ml Vinegar with %1 chol**	**Normal diet**
glucose	baseline	*64.8 ± 17.4*	87.5 ± 20.9	80.2 ± 13.2	91.75 ± 5.7
	postprandial	*122.8 ± 16.4*	102.6 ± 22.1*	124.8 ± 19.8	92.65 ± 7.9*
TC	baseline	61.8 ± 12.1	71.2 ± 15.7	96.2 ± 33.8	97.8 ± 23.7
	postprandial	92.8 ± 40.4	95 ± 31.3#	51 ± 7.7*	89.5 ± 19.4
LDL-C	baseline	32.5 ± 12.2	38.7 ± 12.4	57 ± 26.5	49.3 ± 22.5
	postprandial	44.8 ± 30.5	49 ± 24.9#	22.5 ± 4*	44.8 ± 18
HDL-C	baseline	16.7 ± 3	20 ± 6.2	23.7 ± 9.4	25.3 ± 4.5
	postprandial	14.33 ± 4.08	19.8 ± 2.3	15.8 ± 5.3	19.5 ± 4.2
TAG	baseline	148.7 ± 62.9	166.7 ± 40.4	166.5 ± 73.2	149.5 ± 56.2
	postprandial	164 ± 54.6	167 ± 70.1	148 ± 44.7	166.3 ± 61.1
ApoA1	baseline	20.8 ± 2.5	22.2 ± 4.8	24.5 ± 4.7	34 ± 2.4
	postprandial	19.08 ± 2.42	26.6 ± 3.4	21.8 ± 5.6	29.5 ± 3.5
ApoB100	baseline	5.2 ± 2.5	7.7 ± 3.4	8.2 ± 2.6	4.5 ± 1.9
	postprandial	*5.8 ± 3*	8.4 ± 4.83#	3.55 ± 1*	4.3 ± 2.1

Mean serum glucose, TC, LDL-C, HDL-C, TAG, ApoA1, ApoB100, mg/dl ± SD, in each group (n = 8 for each experimental group).*p < 0.05: Pairwise comparison of differences between baseline and postprandial when compared to cholesterolemic group. # p < 0.05, Mean differences between baseline and postprandial 5 ml vinegar with respect to 10 ml vinegar.

### Serum transaminases levels

No significant difference in serum SGOT and SGPT levels were found between low- and high-dose vinegar with cholesterolemic diet groups compared to hypercholesterolemic diet group (*P *> .05) (Table 2).

**Table 2:** Comparison of serum glutamic pyruvic transaminase(SGPT), serum glutamic oxaloacetate transaminase(SGOT) before (baseline) and after (postprandial) experimental diet

Biochemical factors (u/l)		Groups			
		**Cholesterolemic diet**	**5 ml Vinegar with%1 chol**	**10 ml Vinegar with %1 chol**	**Normal diet**
**SGOT**	**baseline**	62.7 ± 19.7	71.8 ± 36.8	57.5 ± 48.3	56.25 ± 14.7
	**postprandial**	80.4 ± 20.6	61.4 ± 26.1	72.5 ± 23.8	41.5 ± 23.4
**SGPT**	**baseline**	62.8 ± 16.5	70 ± 21.2	58.2 ± 43.1	50.25 ± 4.1
	**postprandial**	74 ± 18.2	70.6 ± 18.6	75.5 ± 30.7	37.3 ± 13

Mean SGOT and SGPT, u/l ± SD, in each group (n = 8 for each experimental group).

### Endothelial markers

The serum level of nitrite in normal diet control was significantly decreased comparison with hypercholesterolemic diet (*P *= .01). Nitrite concentration was decreased in low-and high-dose vinegar with hypercholesterolemic diet groups compared to rabbits fed high cholesterolemic diet, but the difference was not significant(*P *> .05). No significant difference in nitrate concentration was found between low and high-dose vinegar with cholesterolemic diet groups compared to hypercholesterolemic diet group (Table 3).

**Table 3:** Comparison of nitrite and nitrate values before (baseline) and after (postprandial) experimental diet

Biochemical factors(μmol/l)		Groups			
		**Cholesterolemic diet**	**5 ml Vinegar with %1 chol**	**10 ml Vinegar with %1 chol**	**Normal diet**
**Nitrite**	**baseline**	33.78 ± 23.1	31.19 ± 18.4	32.54 ± 32.7	34.49 ± 21.0
	**postprandial**	81.17 ± 28.8	58.27 ± 28.2	62.62 ± 42.0	20.32 ± 3.6*
**Nitrate**	**baseline**	17.9 ± 5.1	19.7 ± 6.9	17.0 ± 2.1	16.7 ± 1.6
	**postprandial**	23.0 ± 8.6	24.1 ± 5.1	20.4 ± 8.7	12.3 ± 3.5

Mean Nitrite and Nitrate, μmol/l ± SD, in each group (n = 8 for each experimental group).*p < 0.05: Pairwise comparison of differences between baseline and postprandial when compared to cholesterolemic group.

### Inflammatory factors

High cholesterol control induced a significantly increase in fibrinogen comparison with low-dose vinegar with cholesterolemic diet (*P *= .01). Though reduced fibrinogen level was found in high-dose vinegar with cholesterolemic diet comparison to hypercholesterolemic diet but the difference was not significant.

In high cholesterol group, CRP was increased significantly compared to the normal diet group (*P *< .0001). No significant difference was found between low and high-doses vinegar with cholesterolemic diet compared to hypercholesterolemic diet. Also, no significant difference was shown between low and high-doses of vinegar (Table 4).

**Table 4:** Comparison of fibrinogen and C-reactive protein(CRP) values before (baseline) and after (postprandial) experimental diet

Biochemical factors		Groups			
		**Cholesterolemic diet**	**5 ml Vinegar with %1 chol**	**10 ml Vinegar with %1 chol**	**Normal diet**
**CRP(μg/ml)**	**baseline**	2.43 ± 0.35	2.37 ± 0.26	2.48 ± 0.32	2.51 ± 0.12
	**postprandial**	3.72 ± 0.20	3.42 ± 0.19	3.46 ± 0.16	3.02 ± 0.27*
**fibrinogen (mg/dl)**	**baseline**	195.4 ± 17.2	227.6 ± 51.9	209.17 ± 20.3	240.75 ± 16.8
	**postprandial**	256 ± 35.3	221.5 ± 53.3*	228.83 ± 38.3	257.75 ± 23.3

Mean CRP and fibrinogen, μg/ml ± SD and mg/dl ± SD, respectively, in each group (n = 8 for each experimental group).*p < 0.05: Pairwise comparison of differences between baseline and postprandial when compared to cholesterolemic group.

### Oxidative factors

MDA increased significantly in hypercholesterolemic diet comparison with normal control diet (*P *= .001). Consumption of high-dose vinegar with cholesterolemic diet induced a significantly decrease in MDA comparison with hypercholesterolemic diet (*P *= .031). No difference significant between low and high doses of vinegar.

Significant difference was observed between hypercholesterolemic diet compared to normal diet by ox-LDL (*P *= .035). Using high-dose vinegar with cholesterolemic diet induced a significant decrease in ox-LDL compared to hypercholesterolemic diet (*P *= .020). The difference between low and high-doses vinegar was not significant (Table 5).

**Table 5:** Comparison of oxidative factors values before (baseline) and after (postprandial) experimental diet

Biochemical factors		Groups			
		**Cholesterolemic diet**	**5 ml Vinegar with %1 chol**	**10 ml Vinegar with %1 chol**	**Normal diet**
**MDA (mol/l)**	**baseline**	1.42 ± 0.37	1.4 ± 0.29	1.35 ± 0.32	1.28 ± 0.20
	**postprandial**	2.88 ± 0.71	2.25 ± 0.46	2.04 ± 0.40*	1.18 ± 0.18*
**OXLDL(ng/ml)**	**baseline**	*32.99 ± 11.2*	30.05 ± 6.3	33.38 ± 18.8	24.08 ± 6.6
	**postprandial**	69.59 ± 19.4	54.25 ± 32.0	52.41 ± 28.2*	34.62 ± 7.6*

Mean MDA and OXLDL, mol/l ± SD and ng/ml ± SD, respectively, in each group (n = 8 for each experimental group).*p < 0.05: Pairwise comparison of differences between baseline and postprandial when compared to cholesterolemic group.

## Discussion

Concomitant consumption of cholesterol enriched diet with vinegar modifies the atherogenic effects of cholesterol and significantly prevents the increase of ox-LDL, MDA, LDL-C, ApoB100, TC, glucose and fibrinogen.

Food intake is generally followed with a raised level of triglyceride and glucose as well as increased oxidative reactions. In postprandial state, cellular structures such as proteins, carbohydrates, nucleic acids and lipids are damaged by oxidative processes [27]. Some recent studies [1,28,29] have suggested that a high fat diet including TG rich lipoproteins plays a role in initiation of atherosclerosis since it can cause an acute modification in endothelial function. This effect begins two hours after food intake and continues for several hours. It is supposed that raised postprandial TG and cholesterol levels are mainly responsible for this effect. Using low-dose vinegar with cholesterolemic diet induced a significant decrease in serum glucose level compared to hypercholesterolemic diet. Results of Johnston CS et al indicated that vinegar and peanut products as complementary foods to reduce postprandial glycemia [16]. The antiglycemic property of vinegar might be due to the inhibitory effect of acetic acid on some enzymes of metabolism carbohydrate such as sucrose, lactase and maltase activities during the posttranslational processing [30]. Delayed gastric emptying might also contribute to antiglycemic effect of vinegar [31].

In case of lipid profile, we found that high-dose vinegar can make a significant acute decrease in TC, LDL-C concentrations and obviously it was much more effective than 5 ml vinegar. According to the results of Fushimi, Sato et al, acetic acid reduced malonyl-CoA in the liver of rats under postprandial condition [32]. Since the activation of AMP-activated protein kinase (AMPK) (inhibitor of fatty acid and sterol synthesis) leads in the reduction of malonyl-CoA content [33], thus consumption of acetic acid can influence lipid synthesis. Daher et al found a decreased plasma level of TG and chylomicrons concomitantly with delayed gastric emptying three hours after ingestion of pineapple and grapefruit in normolipidemic rats [21].

Apolipoproteins such as ApoB are known as significant predictors of development atherosclerosis and even better than lipid profile [34].

Using high-dose vinegar induced a significant decrease in ApoB100 compared to hypercholesterolemic diet. Decrease of ApoB suggests an effect on lipoprotein production, such as a delay in fat reabsorbtion and or a decrease in the secretion of the particles from entrocytes. Studies conducted on the components of vinegar have shown the presence of high amounts of polyphenols, vitamin C and organic acids [10,11]. Juhel et al suggested a mechanism by which polyphenols may decrease fat digestion and absorption in gastric. They indicated that lipid emulsification process in gastric and pancreatic lipase activity in gastric and duodenal media is inhibited by cateching-rich polyphenolic [35,36]. Polyphenols have also been shown to play a role in the controlling of key intracellular enzymes contributed in the synthesis and secretion of ApoB-containing lipoproteins [37,38]. ApoA, which has a protective role in cardiovascular diseases was increased in vinegar taking groups specially 5 ml vinegar (*P *> .05). Using high-dose vinegar was significantly better than low dose vinegar in decreasing ApoB100 level (*P *< .05). The effect of acute red wine polyphenol consumption on postprandial lipaemia in postmenopausal women studied by Naissides M et al and they concluded that dealcoholised red wine (DRW) consumption did not affect on postprandial TG level but, qualitatively, after 6 hours DRW can reduce the exposure of arteries to lipoprotein containing apoB48 [39].

Recently in several studies the association of liver transaminases with some components of metabolic syndrome has been reported. It has been supposed that an increasing SGOT/SGPT ratio can predict coronary atherosclerosis [40]. However, we did not find any significant acute effect of vinegar on serum transaminases.

Nitric oxide (NO) is the most important endothelium derived vasodilator. In our study, nitrite increased in high cholesterol control compared to normal diet. Both low and high-doses vinegar with cholesterolemic diet showed a reduction in nitrite compared with high cholesterol control, however the difference was not significant. Pergola et al investigated the effect of anthocyanin fraction of black berry extract on nitric oxide and found that anthocyanin can suppress NO production through inhibition of expression and activation of inducible nitric oxide synthase (iNOS). In particular, the protein expression was inhibited through the reduction of NF-κB and/or mitogen activated protein kinase (MAPK) activation [41]. The same mechanism might be considered for vinegar in lowering NO metabolites. The acute and chronic effects of drinking alcoholic beverages at dinner on serum nitrate and nitrite (NOx) was studied by Sierksma A et al [42]. They indicated that though an acute and transient increase of NOx would be observed following food intake, but the concomitant use of alcoholic beverages would decrease the effect slightly.

No significant difference was found between vinegar taking groups and high cholesterol control in CRP. In high cholesterol group, CRP was increased significantly compared to the normal diet group. Inflammatory process and thrombosis/fibrinolysis system investigated by Tousoulis et al and no significant change of inflammatory factors was shown in any of the studied groups [43]. In study performed by Alexopoulos et al on the acute effect of green tea consumption on endothelial function in normal subjects, they measured CRP at baseline and at 120 min after consumption green tea and caffeine and results of their studies showed that tea and caffeine had no effect on CRP [44].

We may conclude that vinegar does not affect acutely on CRP but future studies should investigate chronic effects and also consider other markers of inflammation.

We found that low-dose vinegar appeared to be effective in reducing plasma fibrinogen concentration (*P *< .05). Though reduced fibrinogen level was found in high-dose vinegar comparison to hypercholesterolemic diet but the difference was not significant.

According to the results of Grenett HE et al polyphenols (catechin and quercetin) may have cardioprotective properties due to alterations of fibrinolytic protein mRNA expression and the resultant increased fibrinolysis of endothelial cells [45].

MDA as a thiobarbituric acid reactive substance is the end-product of lipid preoxidation. Considering the cytotoxicity and genotoxicity of lipid oxidation end products (ALEs) like oxycholesterol, 4-hydroxy-nonenal, and malondialdehyde, consumption of high-fat foods containing ALEs is relatively responsible for atherosclerosis [46,47].

We found that in high-dose vinegar MDA level decreased significantly compared to cholesterol rich diet and this acute effect means that flavonoid contents of vinegar might be effective in reducing lipid peroxidation and enhance the antioxidant enzyme activity [48]. Reduction in atherosclerosis by antioxidants has been related with a decrease in reduction MDA [49,50]. Several studies indicated that flavonoids can prevent lipid peroxidation in microsomes and liposomes. The antioxidant potency of flavonoids in these studies depends on the arrangement of hydroxyl groups on the benzene ring [51-54].

Gorelik S et al investigated effect of red wine on postprandial MDA. The results revealed a relatively rapid accumulation of MDA in plasma, with a maximum level achieved 3 h after the meal. They showed that red wine prevent absorption of the lipotoxin MDA [55]. Further studies should be focused on antioxidant potential of vinegar and the mechanisms.

The ways postprandial hyperlipemia might induce its atherosclerotic effects have been studied by Natella et al [55]. As a probable mechanism, increased postprandial level of plasma lipid hydroperoxides (LPO) and the resultant increased susceptibility of LDL to oxidation were suggested. This phenomenon is mediated by LPO-induced effects on oxidant/antioxidant balance [56].

As expected, using high-dose vinegar with cholesterolemic diet induced a significant decrease in ox-LDL compared to hypercholesterolemic diet. The mechanism by which flavonoids inhibit LDL is not totally known, but it is supposed that they decrease free radical formation, protect LDL-α-tocopherol or regenerate oxidized LDL-α-tocopherol, and/or separate metal ions which contribute in oxidation reactions [57,58].

Studies indicated that the phenolic compound contents of red wine [59] and walnuts [60] can lower oxidation LDL after acute consumptions of these foods. According to the results of Natella et al using grape seed proanthocyanidins with meal can lower the postprandial oxidative stress through decreasing oxidants concentration and increasing the level of serum antioxidants. These effects can eliminate the oxidative modification of LDL [55].

Kondo et al demonstrated that 2 hours after consumption of 35 grams of defatted cocoa enhanced the resistance to oxidative modification of LDL [61]. Several studies suggest that postprandial lipemia increases risk of atherogenesis, thus assessment and treatment of atherosclerosis should include parameters associated to postprandial lipemia. Diets high in fat is contributory risk factors, whereas consumption of polyphenol-rich fruits and vegetables during the meal seems to reduce these risk factors. The results of this study show that vinegar consumption with hypercholesterolemic diet decrease destructive effects of a cholesterol rich diet. Different doses of vinegar show different effects on biochemical factors. Low-dose vinegar affect on fibrinogen and glucose and high-dose vinegar has significant effect on MDA, ox-LDL, TC, ApoB, and LDL-C. Therefore it seems that higher dose affect on lipoproteins, apolipoproteins and oxidation. Further effort is needed to improve the reliability of these results and to shed light on underlying mechanisms. Also future researches must focus on chronic effects of vinegar intake. Although the present data do not allow the conclusion that vinegar intake has an acute protective value for atherosclerosis, it seems reasonable to conclude that the results can probably show the favorable acute effects of vinegar on some of the biochemical risk factors of atherosclerosis. Regarding the availability and affordability of vinegar, these findings indicate the potential for vinegar to be considered in prevention of some of the risk factors of atherosclerosis. Future studies must focus on determining similar effects of vinegar on human.

## Competing interests

The authors declare that they have no competing interests.

## Authors' contributions

MKH participated in the sequence alignment and drafted the manuscript, NE carried out the laboratory tests. AE participated in the sequence alignment. MS participated in the design of the study and performed the statistical analysis. AHR conceived the study, and participated in its design and coordination. All authors read and approved the final manuscript.
